# A Structured Approach for Investigating the Causes of Medical Device Adverse Events

**DOI:** 10.1155/2014/314138

**Published:** 2014-11-27

**Authors:** John N. Amoore

**Affiliations:** Department of Medical Physics, Crosshouse Hospital, Kilmarnock KA2 0BE, UK

## Abstract

*Aim*. Medical device-related adverse events are often ascribed to “device” or “operator” failure although there are more complex causes. A structured approach, viewing the device in its clinical context, is developed to assist in-depth investigations of the causes. *Method*. Medical device applications involve devices, clinical teams, patients, and supporting infrastructure. The literature was explored for investigations and approaches to investigations, particularly structured approaches. From this a conceptual framework of causes was developed based primarily on device and clinical team caring for the patient within a supporting infrastructure, each aspect having detailed subdivisions. The approach was applied to incidents from the literature and an anonymous incident database. *Results*. The approach identified and classified the underlying causes of incidents described in the literature, exploring the details of “device,” “operator,” or “infrastructure” failures. Applied to incident databases it suggested that causes differ between device types and identified the causes of device unavailability. *Discussion*. The structured approach enables digging deeper to uncover the wider causes rather than ascribing to device or user fault. It can assess global patterns of causes. It can help develop consistent terminology for describing and sharing information on the causes of medical device adverse events.

## 1. Introduction

Most clinical applications of medical devices are safe and effective, but occasionally adverse events do occur [1–7]. Typically the adverse events involving medical devices have several causes, with human fallibility (to err is human [8]) combining with technological imperfections (design, usability, or reliability) and limitations in the supporting infrastructure (maintenance, utility supplies, and procurement processes) to cause incidents. A full understanding of the often complex causes requires a systems approach [1, 9]. Furthermore, adverse events are investigated not simply to retrospectively analyse what went wrong, but to learn lessons to prevent repetitions [10]. Learning lessons requires a comprehensive understanding of the causes. A holistic systems approach to investigations can help ensure that the underlying causes are understood, in turn supporting the development of safety nets and barriers (redesign, alarms, monitoring, procedures, and user interventions) that can help prevent recurrences [9].

Despite the well-recognised multifactorial causes of medical device-related adverse events many investigations focus on a “device” or “user” error approach. This narrow often subconscious “device-or-user” focus can blind investigators, preventing them from understanding the details of what went wrong, whether of any “device” or “user” failure, and details of any system interactions involving human factors that may have been involved [11, 12]. It is thus perhaps not surprising that the cause is often not established [6].

A structured approach can clarify the nature of the causes as shown in an investigation of the causes of medical devices adverse events occurring in operating theatres [13]. A review of operating theatre adverse events found that over 20% were equipment related. The structured approach showed that many device-related failures were caused by equipment configuration and setting (43%) or unavailability at the point of need (37%), with only 34% due to device malfunction (34%).

Whilst the “device-or-user” approach has often been adopted, systematic approaches to investigate the causes have been developed. Marvin Shepherd [14] defined five failure types or groups: equipment, operator, facility (e.g., electrical supply), environment (e.g., electromagnetic interference), and patient (Table 1). Each of these groups is subdivisible to dig deeper into the cause. The “operator” group, for example, includes “training”, “use”, “diverted attention,” and “criminal intent.” The system's strength consists in its division of the root causes into readily understandable broad groups, with the subdivisions supporting detailed analysis where appropriate, dependent on investigation requirements and available data. The 5 main groups encourage the investigator to consider causes more widely than simply device or operator, with the subdivisions of each group helping to understand the details. The hierarchical approach of broad groups that can be subdivided has also been adopted by the ECRI Institute, which similar to the Shepherd approach divided causes into 5 broad groups though with slightly different groups: equipment factors; external factors; support system failures; tampering and/or sabotage; and user errors (Table 2) [7].

A hierarchical approach to classify the causes was also adopted by the International Standards Organisation [24, 25]. It was developed primarily to facilitate data sharing between manufacturers and regulatory authorities, but the introduction also suggests that it can be applied as part of healthcare reporting systems. The coding structure is based on 30 event types such as “Activation, Positioning or Separation” (e.g., failed patient lead connection); Computer Hardware; Computer Software; electrical/electronic (e.g., circuit board failure, battery); External Conditions (inadequate room ventilation, failure of external power source); “Marking, Labelling or Instructions for use”; Mechanical; and Unintended Function and User Error. The titles of these event types illustrate that they have been developed from technical rather than clinical perspectives. Each event type can be explored in more detail using subdivisions as illustrated for some above. The term “Use Error” is frequently used when discussing the causes of adverse events. In the ISO system it is an event type whose subdivisions include Inadequate Disinfection, Inadequate Training, Maintenance, Device Inoperable, and “Use of Device Issue.” This latter subdivision is defined as being associated with the “failure to process, service, or operate the device according to the manufacturer's recommendations or recognized best practices.” The “Unintended Function” event type addresses devices “not working as intended” with subdivisions including the device displaying incorrect information and delivering energy to the incorrect body part. An annex has useful examples of applying the codes, showing that adverse events typically are associated with more than one cause. The event codes are integrated with a holistic coding structure that includes device type and patient outcome.

Medical device adverse event investigation is multidisciplinary and requires terminology and an approach that is readily understandable by the clinical and technical staff involved. The approach adopted should guide those reporting the event to clearly describe it and any initial thoughts on the causes. The approach should also support those investigations to uncover and identify the underlying causes. It should be flexible to adapt to developments in healthcare and technology. It should be based on clear concepts with meaningful and useful categories that are applicable to all healthcare settings [26].

## 2. Methods: Developing a Structured Approach

A structured approach to investigate adverse events was developed by considering how medical devices are used in healthcare and by critically reviewing the hierarchical systems described earlier [7, 14, 24, 25].

Medical devices are used by professional and, increasingly, lay staff to support and deliver care. The application involves interactions between the device, the clinician/carer, and the patient. Its application occurs within an environment that includes physical and supporting infrastructures. This will generally include a physical building and often supplies of electrical power and perhaps medical gases. It will also include the physical mounting and position of the devices. Less obvious are supporting systems that help ensure the availability of the equipment and its consumables and accessories (e.g., procurement processes), training support for users, and maintenance arrangements to ensure that the equipment continues to operate safely and reliably.

**Figure 1:**
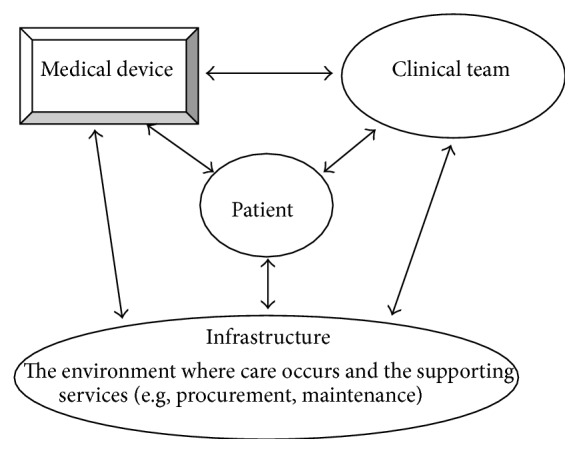
Diagram summarising the interactions between a medical device, the clinical team, and the patient within an infrastructure that includes both the physical environment and the supporting services. Each of the elements (device, clinical team, patient, and infrastructure) interacts and depends on each other.

Figure 1 summarises these elements and the interactions between them. The term “Clinical Team” refers to the professionals (doctors, nurses, and allied healthcare professionals) and, in the community, lay carers looking after and managing the patient. Medical Device includes the device itself and its associated accessories (such as patient leads) and consumables. The interactions may be direct and explicit, as, for example, the physical connection between an infusion pump or a patient monitor and the patient. Some interactions are neither tangible nor explicit; for example, the supporting systems that ensure that the correct consumables are available for the medical device or, for example, the maintenance and support systems that ensure that the equipment is operating reliably, safely, and effectively.

Figure 1 suggests four main elements that are involved in safe operation of the medical devices: the device itself, the operator, the patient, and the infrastructure within which the care takes place. Failures or operational problems in any of these elements, or in the interactions between them, can lead to an adverse event. An interaction that goes wrong may be, for example, clinical staff incorrectly setting the controls for respiration rate on a ventilator. An interaction that is perhaps less obvious is a procurement process that delivers the wrong consumable for the medical device, causing a malfunction that harms the patient.

This systems approach suggests that a structured investigation approach should incorporate at the least these four elements. But the approach needs greater depth, a greater understanding, and recognition of what aspects of each element could contribute to an adverse event. What is involved in the failure of any of the elements, for example, of operator error, device failure, or infrastructure failure? Citing the cause as simply “device” does not sufficiently describe the reason nor begin to learn how to prevent repetitions. “Device” failure may be caused by poor human factors design [3, 5, 11, 12, 27], poor technical design, poor manufacture, or random component failure. Similarly, citing the failure as “operator,” “infrastructure,” or “patient” only begins to understand the causes. The approach must provide for and encourage deeper, detailed questioning.

In some cases there may be a less clear-cut distinction as to whether a particular failure is ascribed to one or more of the elements, for example, as to whether the failure is due to an operator error or an error resulting from poor device ergonomics. Poor design, particularly of devices used in complex stressed clinical situations, can predispose to adverse events [3], which even detailed operator training cannot prevent [27, 28]. This distinction does not imply that competency staff training is not important nor does it imply that operators do not make mistakes; they do, as exemplified in the phrase “to err is human” [8]. Rather the distinction seeks to explicitly show that poor design can cause adverse events. This distinction is supported by the ISO classification [24, 25] that distinguishes between “usability,” namely, “deficient usability causing device failure” and “use error,” that is, “an act or omission of an act that has a different result than that intended by the manufacturer or expected by the operator.”

Instances of poor ergonomic design, poor device usability should be treated as a device error as, for example, the “human factors design” failure described by Shepherd (Table 1 [14]). However, there will be occasions where the cause was an act or omission of an act by an operator, in which case the fault should be described as an example of an “operator” failure.

Distinct from ergonomic failures are technical design failures. Other device failures include random component or subassembly failure, battery failure, wear and tear, and chemical damage. It is important that the consumables and accessories used with medical devices are not overlooked. The use of faulty consumables and accessories can cause adverse events.

The term “operator failure” covers several failures, an understanding of which can be assessed by reviewing the process in which an operator deploys a medical device, starting with selecting the appropriate device, setting it up (including attaching accessories and consumables), setting the controls and alarms, performing preuse checks, and ensuring safe and effective performance during its operation.

The structured approach must accommodate failures arising from not following approved operating procedures and instructions for use, as well as accommodating adverse events caused by faulty operating procedures. Before using a medical device operators have a personal responsibility for ensuring that they have attained the understanding and competency training recommended by the manufacturer of the device. Where an operator fails to follow procedures then the approach should enable this to be described as an aspect of an operator error. However, where the procedures are flawed then the cause should be able to be ascribed to faulty operating procedures. This could be a subset of a “device failure” if the faulty operating procedures or instructions for use were developed by the manufacturer or the supplier. However, if the flawed operating procedures were developed by the healthcare organisation, then the failure should be ascribed as an aspect of an “infrastructure” failure. “Operator failures” must include provision for distraction and fatigue in the often busy clinical environment. Finally, although rare, provision must be made as a cause for deliberate or malicious misuse.

“Infrastructure” has a broad meaning, incorporating the immediate physical clinical environment where healthcare takes place and the background infrastructure and support systems (including maintenance). Basic infrastructure problems may include failures in supply of electricity or medical gases or electromagnetic interference. Often overlooked, but a significant cause of adverse events, is device unavailability at the point of clinical need [11, 13]. Unavailability is an infrastructure failure, recognising the organisation's responsibility for the provision of safe and effective medical devices at the point of need. There are many reasons for lack of availability including lack of procurement, failures of resource management, housekeeping, and delayed maintenance [29]. Poor environmental ergonomics, including device layout, can contribute to incidents, perhaps associated with difficulties accessing controls or seeing displays. Poor mounting can cause devices to fall, perhaps onto patient or staff. Faulty maintenance contributes to incidents as does poor installation and commissioning.

The detailed components of the device, operator, and infrastructure elements of Figure 1 need to be included in the structured approach. The three hierarchical systems described earlier, Shepherd [14], ECRI [7], and the ISO classification [24, 25], include subgroups enabling the causes to be described in greater depth. Both Shepherd and ECRI included device and operator. Shepherd included both “facility” and “environment” groups, with the latter described as internal or external to the hospital (Table 1). ECRI described an “external” group that included utility supplies and electromagnetic interference and several “support system failures” (Table 2). The schematic diagram of Figure 1 suggests incorporating both ECRI's “external” and “support system” groups and Shepherd's “facility” and “environment” groups into a single “infrastructure” group. It is suggested that this may be easier for investigators to understand. The “infrastructure” group will be designed to include the appropriate elements from Shepherd's and ECRI's classifications, arranged in logical subdivision.

The ISO classification system [24, 25] has many positive features. It is a standard that has obtained recognition and is designed as a hierarchical system. However, it has been designed from a technical perspective rather than from a perspective that is readily understandable by clinicians in healthcare.

The detailed subdivisions are developed from reviewing classification systems [7, 14, 24, 25] and by analysing adverse incidents described in searchable databases and published reports [1, 3, 4, 15]. The subdivisions can be extended within the hierarchical structure as healthcare and its technology develops. Subdivisions should be logical, for example, with the device subdivisions following the device pathway from design to operational use. Similarly infrastructure subdivisions start with the procurement and commissioning and work through the support required during operational use.

Table 3 presents the hierarchical structure, developed from Figure 1, which supports an open-minded logical approach to investigation. Provision is required for “unknown” where an investigation concludes without cause identification. On the other hand the investigation might conclude that no incident actually occurred (no problem found). The cause might also be deliberate, either malicious or misguided tampering by patient or visitor, as provided for by the “tampering” of the ECRI classification as shown in Table 2. Shepherd [14] included provision where the adverse event was caused by clinical and patient factors where interactions between device and patient condition cause adverse effects (Table 1) and provisions for this type of event have been included.

The investigation approach is illustrated by applying it to incidents selected largely from the literature, with some anecdotal examples, none from the author's local hospitals. Each incident is summarised and analysed with the causes identified using the schematic diagram of Figure 1 and its related table (Table 3). An anonymous database of incidents was retrospectively analysed to show how the cause-grouping can reveal trends and global causes of adverse events.

## 3. Results

Table 4 summarises the application of the approach to incidents described in the literature. The table details the causes with the main group in italics, followed by subdivision details. The details in the subdivisions depend on the information available about the incident. The analysis reveals that most incidents have several causes. The analysis can also reveal protective barriers and positive steps taken to minimise incident consequences; examples are shown in the “Good Practice” column.

**Figure 2:**
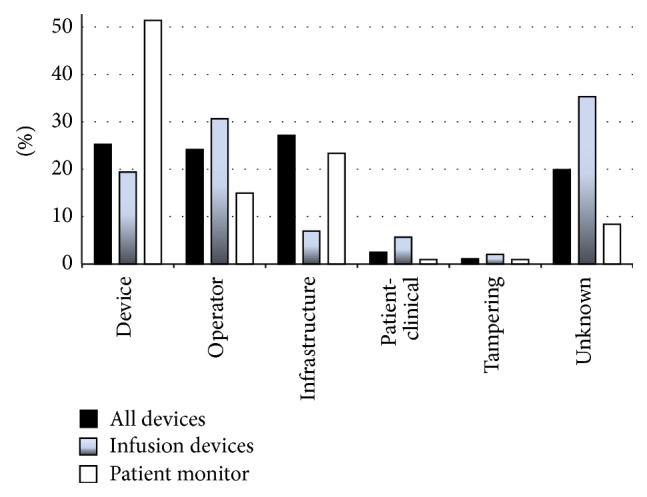
The causes of medical device adverse events can differ dependent on medical device type. The graph shows the distribution of the 6 causes shown for all the incidents, shown separately for all the devices (as a percentage of the 1619 causes identified), for the infusion devices (as a percentage of the 623 causes identified), and for the patient monitors (as a percentage of the 107 causes identified).

**Figure 3:**
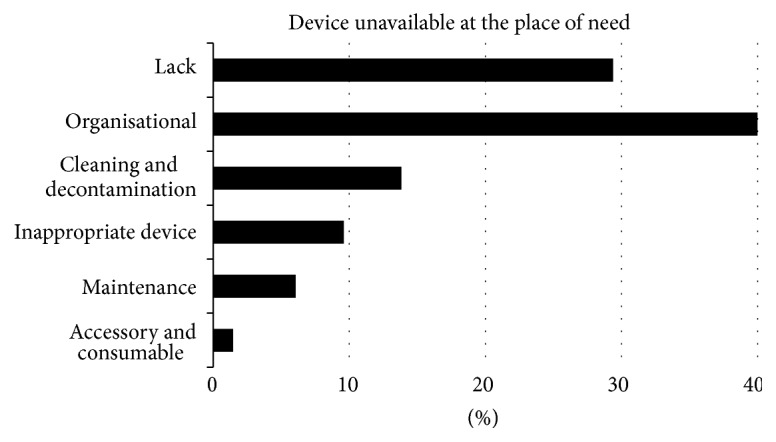
What causes medical device unavailability at the place of clinical need? The data are expressed as percentages of the 283 instances of lack of availability of medical devices.

The approach was used to analyse an anonymous database of 1404 incidents (not from the author's own institution).  Figure 2 illustrates that the causes may differ between device types (with details on infusion devices and patient monitors illustrated). The prevalence of the causes is expressed as percentages of the number of causes identified for each device type: of the 1619 causes identified for all the device types, of the 623 causes of infusion device incidents, and of the 107 causes of patient monitor incidents. More than half the patient monitor incidents were identified as being caused by device failures, whilst the cause was not identifiable in over 40% of infusion device incidents, with most of the remaining being caused by operator failure. Device, operator, and infrastructure causes were more evenly distributed across all the medical device incidents in the database. The structured approach was also applied to investigate the reasons for the unavailability of medical devices at the point of clinical care (Figure 3). In the example database studied 17% of the incidents were attributed to unavailability of medical devices, with analysis suggesting that in many cases the unavailability was caused by organisational aspects rather than an absolute lack.

## 4. Discussion

A structured approach based on the interactions that occur when medical devices are used in healthcare has been developed to support investigating the causes of adverse events. Building on previous work [7, 14] the development was guided by principles of clear meaningful concepts applicable across healthcare settings [26]. It considers technologies role in healthcare with the patient the focus, cared for by people (professional and increasingly lay carers in the community), supported by technology within an environment of care. The environment could be a tertiary academic hospital or the bedroom in a patient's home. The approach was designed to be applicable to all care settings, not limited by technology type, clinical specialism, or type of healthcare. It groups the causes based on a schematic diagram of patient care situations (Figure 1). The development explores these groups in depth, identifying their characteristics and incorporating them into the approach as subdivisions within the hierarchical framework provided by the broad groups (Table 3). The approach supports multidisciplinary investigations, avoiding terminology and jargon associated with a particular profession, technical or clinical.

The framework creates a logical hierarchical structure that is designed to encourage investigators to dig deeper, for example, not simply ascribing causes to device or operator, but clarifying the device or operator aspects involved. Exploring in greater detail may help reveal the latent factors and events that primed the incident's trigger. In turn discerning the latent factors may suggest methods that can be put in place to prevent or to minimise risks of recurrence. The subdivisions listed in Table 3 are not exhaustive, but as the nature of the causes are better understood, additional details can be added to within the logical framework.

The approach can be applied to individual incidents where it can help explore the complex nature of the underlying causes as illustrated in Table 4. Guided by the structured approach (Figure 1 and Table 3) multiple causes associated with a particular incident can be explicitly identified, revealing the multiple layers that Reason described diagrammatically in his Swiss cheese model [9]. By identifying the contributory factors the approach lends itself to progressing from cause identification to cause prevention, suggesting processes that can be applied to fill in the holes of the Swiss cheese or to rearranging the layers and their placements to prevent alignment of causes.

The approach can also be used to review global trends in databases of adverse events.  Figure 2 shows, for example, its application to identify, in the incident database investigated, whether the causes differ between device types. This can concentrate remedial action appropriately for different device types, particular when the investigation is deepened to explore in detail the nature of device, operator, or Infrastructure aspects. The identification of the underlying causes using a structured consistent approach can facilitate global understanding of the causes. For example, Figure 3 illustrates how a global analysis can be used to examine why devices are unavailable at the point of need.

The approach has been applied retrospectively to incidents to assess its ability to identify and describe the causes of adverse events. Retrospective analysis of incidents has limitations, partly because of the limited information contained in the summary descriptions of incidents and partly because of the inability to enquire more deeply into the incident and its underlying causes. The retrospective analysis was carried out to test the approach. Its application described here suggests that it can be used to describe, in a consistent logical manner, the causes of adverse events involving medical devices. It is suggested that the approach should now be applied systematically to assess the causes of adverse events.

The approach extends the classifications developed by Shepherd [14] and ECRI [7]. It combines Shepherd's “facility” and “environment” or ECRI's “external” and “support system failures” groups into a single “infrastructure” group. Combining these aspects into a single, readily understandable concept emphasises the importance of considering contributions of the supporting infrastructure when investigating adverse incidents. For example, Case 11 [23] in Table 4 illustrates an incident with several causes. The incident was partly caused by an operator error, but the operator error was itself partly caused by the layout of the medical devices that prevented the operator clearly seeing the controls—hence identified by the approach as an infrastructure-device layout factor. The approach also shows that the device's human factors design (subgroup of device), in particular the layout of its controls, was also a latent cause. The causes uncovered in these in-depth investigations should prompt managers to review operating conditions, those who procure equipment to include mistake-proofing considerations in their selection criteria and manufacturers to improve device ergonomics.

The approach complements the ISO classification system [24, 25] that was developed primarily for sharing information between manufacturers and regulatory authorities. Its “event codes” concentrate on technical issues but do include user aspects. “Use Error” is an “event code” whose subdivisions include inadequate disinfection, inadequate training, procurement, maintenance, “use of device issue,” and “device inoperable.” Its inclusion of maintenance and procurement as subdivisions of “Use Error” may be appropriate from the manufacturer's perspective (the device is in use rather than in manufacture or design), but not viewed within the context of its clinical application. It is suggested that these are better placed within the infrastructure section as in Table 3.

The Medicines and Healthcare Products Regulatory Agency (MHRA), the competent authority for medical devices in the UK, has started to use the ISO classification system when summarising adverse events reported to it [17]. The report found that “use of device issue” was implicated about 300 times, but well below more frequent causes such as “material separation” (over 1200 times) and “incorrect or inadequate result” (over 800 times). The event “patient-device incompatibility” was cited nearly 800 times; this is defined as being associated with the interaction between device and the patient's physiology or anatomy. The high frequency of this event type in the MHRA report [17] may reflect the number of reported incidents involving implants. The range of causes in this MHRA report illustrates how the findings of global investigations will depend on the nature of the incident group examined. The findings from an analysis of the causes of incidents encountered within an operating theatre environment [13] are likely to be different from those encountered in community healthcare. The structured approach advocated here could be used to investigate the nature of the causes in each of these different areas, recommending appropriate interventions and preventative measures for each.

Whilst the database of incidents analysed as an example in this paper did not include implants the approach does include provision for the interactions between device and patient in the “clinical and patient factors” (Table 3). Alternatively, if the underlying cause was attributed to systematic biological patient-device incompatibility, the cause may be assessed as inadequate design consideration of the biochemical environment of the implant and hence as a device design error. However, a different type of device-patient interaction also requires inclusion as a possible cause. This is needed to accommodate those incidents where patients, confused as a direct result of their medical care (e.g., sedation or medication) can “awake” and pull at devices connected to them—causing damage. This is catered for in the clinical and patient factors.

It is sometimes difficult to differentiate whether the cause is operator failure or poor human-factors design. The “Use Error” event subcodes of the ISO system [24, 25] include both system support causes (disinfection, maintenance, and refurbishment) and operator error (“use of device issue,” namely, the user's failure to operate the device in accordance with the manufacturer's instructions). Including these conceptually different cause types under one umbrella “use error” term can lead to confusion as to what it means. It is suggested that it would be more appropriate to differentiate between maintenance and refurbishment under the “infrastructure” support group with the “use of device issue” term replaced by “user error” and reserved for instances where the cause was identified as a user error.

It is important to differentiate between “use errors,” often as a consequence of poor ergonomic design, whilst restricting the term “user error” to where the operator caused or partly caused the incident. It is recognised that the use of the term “user error” has a negative blame connotation; this is unfortunate as the attribution of the cause to a user error is not aimed at blaming. Rather it is to recognise that operators do make mistakes from time to time. Where the details of the “user error” are known, the nature of the failure to operate correctly should be described—for example, in Example 3 in Table 4. In some instances the details of the operator error may not be known and hence it is important to include a term that recognises that operator failure in general can occur to identify and to develop methods that prevent future occurrences. This may include training which can help ensure safe and effective use. However, it must be recognised that training alone will not prevent operator-associated adverse events [23]. Mistake-proofing [27] should be applied not only to device design (particularly operator controls and displays), but also to procedures and to device mounting and layout. Mistake-proofing, “the use of process or design features to prevent errors or the negative impact of errors” [27] resonates with Reason's [9] barriers and safety nets that prevent errors or omissions from causing harm. Mistake-proofing aims to prevent mistakes and, if mistakes occur, to detect them and prevent adverse consequences. The importance of ergonomics is recognised through the medical device usability standard that requires integration of usability and ergonomics into design [30]. Those who select medical devices should consider usability and ergonomics in their selection criteria.

For global learning and prevention reporting is important [8, 10]. Compiling databases of incidents from healthcare facilities within and between countries can provide a wealth of data whose analysis may reveal patterns and highlight specific problems to be addressed to prevent recurring adverse events. However, effective sharing requires common tools, common descriptors of events and causes, with consistent use of terminology and a structured agreed framework. Consequently, whilst some patient safety classifications have been developed, it has been recognised that “global advances in patient safety have been hampered by the lack of a uniform classification of patient safety concepts” [31]. The work of the ISO is important in recognising the need for a standard and a consistent approach for data sharing for medical device associated adverse events [24, 25]. But more needs to be done to develop terminology and an approach that is intrinsically understandable by clinical and technical staff and increasingly by patients and carers who use and maintain devices.

## 5. Conclusion

A structured hierarchical approach for analysing the causes of medical device-related incidents is presented. Its purpose is to support the development of consistent terminologies for medical device-related adverse event reporting, facilitating the reporting and facilitating the sharing of information on the causes of adverse events, not as an end in itself, but to help improve medical devices, their design, and their safe and effective application [4]. It is based on considering the application of medical devices in healthcare in conjunction with clinical staff within an infrastructure that includes support services. It is designed to facilitate the identification of the multiple causes involved in incidents, aiding investigations by prompting consideration of the wider range of causes. The approach was tested by retrospective application to previously described incidents, but it needs to be tested by those investigating incidents.

## Figures and Tables

**Table 1:** Shepherd's classification of medical device incidents [14].

Device	Operator	Facility	Environment	Patient
(i) Human factors design (ii) Parts/circuit design: unexpected failure (iii) Deterioration: predictable failure that requires preventative maintenance (e.g., battery) (iv) Maintainer error	(i) Education/training (ii) “Use” error (iii) Diverted attention (iv) Criminal intent	(i) Human factors design (ii) Parts/circuits designs: unexpected failure (iii) Deterioration: predictable deterioration that requires preventative maintenance (iv) Maintainer error	(i) Internal to hospital (ii) External to hospital	(i) Active: patient action affected the outcome (ii) Passive: patient's condition affected the outcome

**Table 2:** ECRI classification of medical device incidents [7].

Device	User	External	Support system failures	Tampering or sabotage
(i) Design/labelling error (ii) Device failure (iii) Device interaction (iv) Failure of accessory (v) Improper maintenance/testing (vi) Improper modification (vii) Invalid device foundation (viii) Manufacturing error (ix) Packaging error (x) Random component failure	(i) Abuse of device (ii) Accidental misconnections (iii) Accidental spill (iv) Device misassembly (v) Failure to perform preuse inspections (vi) Failure to read label (vii) Improper connection (viii) Inappropriate reliance on an automated feature (ix) Incorrect clinical use (x) Incorrect control settings	(i) Electromagnetic or radiofrequency interference (ii) Environmental (temperature, humidity) (iii) Medical gas and vacuum supplies (iv) Power supply (including piped medical gas) (v) Water supply	(i) Error in hospital policy (ii) Failure to train and/or credential (iii) Improper storage (iv) Lack of competent accident investigation (v) Lack or failure of incoming and preuse (vi) Poor prepurchase evaluation (vii) Use of inappropriate devices	(i) Tampering

**(a):** 

Device	Operator	Infrastructure
Design (i) Technical design Ergonomics (i) Human factors design Instructions for use (i) Incorrect—faulty (ii) Lack of clarity Manufacture (i) Faulty manufacture Device failure (i) Alarm (ii) Device failure (iii) Software failure (iv) Battery (v) Component failure (mechanical, electrical, subassembly) (vi) Wear and tear (vii) Chemical damage (viii) Biocompatibility System failure (i) Incompatibility between devices (ii) Communication failure between devices IT-medical device (i) Malware attack (ii) Data protection compromised	User error (i) Misuse by operator (user failure to operate correctly) Setting up (i) Inappropriate choice of device (ii) Device assembly (iii) Control setting (iv) Alarm setting (v) Preuse checks not carried out Training from perspective of person providing care (i) Lack of education/training (ii) Knowledge Procedures (i) Failure to follow protocol (ii) Charting, recording (iii) Communication User maintenance (i) Failure to clean (ii) Failure to carry out operator maintenance (iii) Distraction/diverted attention (iv) Fatigue	Procurement and commissioning (i) Procurement of inappropriate devices (ii) Commissioning failure (iii) Lack of training on new device(s) (iv) Lack of communication about new device(s) (v) Delayed commissioning (vi) Installation, including mounting, inappropriate, or faulty Environmental ergonomics (i) Poor ergonomic mounting or layout Utilities (i) Medical gas (ii) Vacuum (iii) Water, including specialised water for dialysis machines (iv) Mains electrical supply Devices unavailable (i) Lack (housekeeping, inadequate supply, lost, stolen) (ii) Appropriate devices not available (iii) Maintenance delays (iv) Existing devices no longer appropriate (v) Procurement installation (lack, delays) Environmental interference (i) Electromagnetic or radiofrequency interference (mobile phone) (ii) Ambient noise
Accessory and Consumable (i) Accessory (ii) Device Consumable	Communicate and concentrate (i) Communicate (ii) Distraction/Diverted attention (iii) Fatigue Criminal intent (i) Deliberate wilful misuse (ii) Malicious misuse	Operating procedures (design and information content of the procedure is faulty) (i) Procedure fault Layout and mounting (i) Ergonomics (ii) Strength and robustness Maintenance (i) Acquisition (ii) Configuration (iii) Inadequate storage (iv) Faulty maintenance (v) Decontamination and cleaning (vi) Failure to upgrade software and/or hardware Network (i) Network failure

**(b):** 

Tampering	Clinical and patient factors	Unknown	No problem found

(i) Patient (ii) Patient on other patients (iii) Visitor (iv) Other	Where interactions between device and patient (including patient's pathology/physiology) contributed to the event	Causes why a conclusion could not be reached: (i) Limited data (ii) No investigation	An incident was reported, but investigation revealed no incident had occurred or no fault was found: (i) No incident (ii) No fault found

**Table 4:** Identifying the causes of incidents using Table 3.

No.	Title	Brief description	Causes	Good practice
1	Burning smell from device “personal communication”	Burning smell from home-use ventilation-assist device. No patient harm. Design: Mains input socket directly soldered to the electronic circuit board, held in place mechanically only by solder connections. Manufacturing process: cutting connector pins short to prevent pins-to-case contact reduced solder-connection surface area. Result: arcing across dry-solder joints that had cracked from flexing between socket and circuit board. Discussions with supplier led to redesign.	*Device*: design—technical design (Mains input socket fixed to the circuit board by solder connections) *Device*: manufacture (cutting pins short)	Prompt reporting by patient and carer Receptive supplier when alerted

2	Overinfusion by syringe pump ([2]; see also [18])	Pump infusing at 15 mL/hr despite rate set at 10 mL/hr. Found during routine check. Pump's damaged syringe size detector incorrectly detected 50 mL syringe as 30 mL causing higher flow rate. Inadequate storage, with pumps piled into a box, may have contributed physical damage exacerbated by fluid ingress corroding the size-detector. Similar incident: missing saddle caused pump to detect as 5 mL a 20 mL syringe [18]. Operator contribution: not confirming correct syringe size at prestart checks.	*Device*: device failure *Infrastructure*: maintenance—inadequate storage *Operator*: setting up-preuse checks not carried out	Regularly checking delivered fluid volume during infusion Prompt staff action

3	Failure to pace heart [2]	Patient's heart paced by external cardiac pacemaker; vital-signs monitor intermittently alarmed asystole. Initially ascribed to either poor intracardiac contact between pacemaker lead and heart muscle or low pacemaker output. Closer inspection revealed insecure connection between pacemaker cable and pacemaker. Design: connector can work loosely. Contributing operator errors: failures to assemble tightly and check connection.	*Device*: device-technical Design (connector type) *Operator*: setting up-device assembly connecting securely *Operator*: setting up-preuse checks not carried out	Use of vital signs monitor Prompt action Open investigation of possible causes

4	Mistaken identity and diagnosis [2]	Patient transfer to coronary care following abnormal diagnostic 12-lead ECG. In CCU heart monitor showed normal ECG. The abnormal ECG was from a different patient. The ECG machine displays the current ECG and stores previous recordings. Instead of printing out the ECG from the current patient, the operator printed out the ECG from the previous recording. Lack of clear print controls contributed to printing wrong ECG. Similar incidents have been reported by NHS England in a patient safety alert [16].	*Device*: ergonomics *Operator*: training—knowledge *Operator*: procedures—charting (not recording patient details on ECG) *Operator*: procedures (not ensuring that ECG matched patient)	Checking diagnosis in CCU before delivering medication

5	Air embolism ([19]; see also [19, 20])	Patient died from air embolism. A cannula, with Luer-Lok connector, was inserted into patient's vein, preparing for intravenous infusion, but not yet connected to infusion pump. Patient's blood pressure (BP) monitored. Hose linking monitor to limb cuff had Luer-Lok connector. Hose disconnected to allow patient to visit bathroom; on return hose was connected to intravenous cannula instead of BP monitor. At next BP measurement monitor pumped air into patient's vein instead of BP cuff. Cannula and cuff had same connectors.	*Device*: ergonomics (similar connectors on cuff and intravenous cannula) *Operator*: setting up—device assembly	

6	Overinfusion [21]	Neonate receiving medication from several syringe pumps. One pump was set to deliver diamorphine at 0.1 mL/hr, but not started. Two minutes later the pump alarmed “attend-to-me.” Not ready to start infusion, nurse stretched to press the alarm-silence button and was reassured by silenced alarm. Later, pressed START, to commence infusion. Neonate suffered massive overinfusion; rate was 10.1 mL/hr − 100 ∗ prescribed rate. When stretching to silence alarm the nurse had inadvertently pressed the “10 mL/hr-increment” instead of “alarm silence” control. Both controls were located close to each other-pressing either silences the alarm.	*Device*: ergonomics (proximity of pump controls) *Infrastructure*: layout—ergonomics *Operator*: setting up—control setting *Operator*: setting up—preuse checks not carried out	

7	Overinfusion ([4], Case 9.10)	To remove air bubbles from the infusion line prior to attaching to patient, the pump's infusion rate was increased to its maximum. After air bubble removal the line was connected to patient. Pump was started without resetting rate-leading to overinfusion.	*Operator*: setting up—control setting *Operator*: procedure (method of priming line [22]) *Operator*: procedure (not checking rate before starting)	

8	Delayed defibrillation ([4], Case 10.5)	Following cardiac arrest defibrillation was attempted. Staff unfamiliarity with defibrillator delayed treatment. New defibrillators had been installed—different type requiring lower energy setting, not consistent with previous protocol. Lack of staff training and comprehensive device commissioning caused operator confusion.	*Infrastructure*: procurement and commissioning—lack of training on new device(s) *Operator*: training	

9	Failure to resuscitate ([15], Case 128950-2009-00277)	Defibrillator unexpectedly switched off during an attempted resuscitation. Battery was 5 years old and had not been regularly checked, contrary to maker's instructions.	*Infrastructure*: maintenance	

10	Overinfusion ([15], Case 01950)	Patient, with pain controlled by patient controlled analgesia (PCA) pump, found with slurred speech. Medication vial was empty, not nearly full as anticipated. Patient had prised the PCA's protective cover to get extra medication. Pump included antitamper protections which had been deliberately tampered with. No system can completely protect against deliberate tampering.	*Tampering*: patient	

11	Overinfusion [23]	Low blood pressure alarm from vital signs monitor-patient receiving medication from infusion pump. Abrupt BP drop resulted from medication free flow from pump. Free flow caused by incorrectly loaded infusion set Incident occurred 3 weeks after new pump's introduction despite intensive operator training that emphasised correct set loading. Pump mounting hindered visibility of misloaded set, exacerbated by staff distraction in busy operating room.	*Device*: ergonomics *Infrastructure*: layout and mounting—ergonomics *Operator*: setting up—device Assembly *Operator*: concentrate—distraction	Prompt staff action
